# Elevated Serum Uric Acid Increases the Risk of Ischemic Stroke Recurrence and Its Inflammatory Mechanism in Older Adults

**DOI:** 10.3389/fnagi.2022.822350

**Published:** 2022-03-08

**Authors:** Han-Yu Zhu, Shu-Zhen Zhao, Meng-Li Zhang, Yan Wang, Zhi-Ming Pan, Hao-Ran Cheng, Ke Zhao, Zhen Wang

**Affiliations:** ^1^Department of Neurology, The First Affiliated Hospital of Wenzhou Medical University, Wenzhou, China; ^2^The First Clinical College, Wenzhou Medical University, Wenzhou, China; ^3^Department of Preventive Medicine, School of Public Health and Management, Wenzhou Medical University, Wenzhou, China; ^4^Department of Neurology, Zhuji Affiliated Hospital of Wenzhou Medical University, Zhuji, China

**Keywords:** UA, ischemic stroke recurrence, NLR, restricted cubic spline regression, mediation analysis, interaction and joint analysis

## Abstract

**Background:**

Serum uric acid (UA) has been reported to be associated with ischemic stroke and inflammation. However, whether or not UA is related to the recurrence of ischemic stroke, and whether inflammation plays a role in the relationship between them remain inconclusive.

**Objective:**

We sought to explore the relationship between UA and the recurrence of ischemic stroke and to define the role of neutrophil-to-lymphocyte ratio (NLR) in the aforementioned relationship.

**Methods:**

A total of 8,995 patients were included in this study. Basic information and blood samples were collected, and whether or not each participant experienced ischemic stroke recurrence within 3 years was documented. Patients were stratified into three groups according to their UA level, as follows: ≤ 266, 267–339, and ≥ 340 μmol/L. COX regression and restricted cubic spline regression models were used to evaluate the clinical correlation between UA and ischemic stroke recurrence, mediation analysis and interaction and joint analysis were used to evaluate the role of NLR in the association of UA and ischemic stroke recurrence, and sensitivity and subgroup analyses were performed to test the robustness of the data.

**Results:**

Ischemic stroke recurrence was related to male sex, older age, higher UA level, higher NLR, hypertension, diabetes, and cardiovascular disease. Following adjustment for potential confounders, a high level of UA (≥ 340 μmol/L) increased the risk of recurrence by 92.6% in patients with previous ischemic stroke. We also found that NLR affects the association between UA and the recurrence of ischemic stroke in older adults, suggesting that patients with high NLR and high UA levels are at greater risk for ischemic stroke recurrence.

**Conclusion:**

UA level is non-linearly associated with recurrence, and NLR has an additive interaction between UA and ischemic stroke recurrence.

## Introduction

Stroke is the second most common cause of death and the third leading cause of disability, with an increasing incidence in developing countries (Campbell and Khatri, 2020). The Global Burden of Disease Study revealed that, although stroke incidence, prevalence, mortality, and disability-adjusted life-years rates declined from 1990 to 2013, the overall stroke burden has increased across the globe in both men and women of all ages (Feigin et al., 2017). Among all types of stroke, ischemic stroke caused by arterial occlusion is responsible for the majority of events (Campbell et al., 2019). Thus, there is a strong argument that we should pay attention to ischemic stroke in particular.

Survivors of stroke and transient ischemic attack are at risk for a recurrent stroke, which is often more severe and disabling than the index event. Recurrent strokes continue to account for 25–30% of all strokes and represent unsuccessful secondary prevention (Hankey, 2014). With recent advances in secondary prevention management, the rate of recurrence has been progressively lowered until the mid-2000s. In the last 10 years, however, the recurrence rate of stroke has not declined further (Flach et al., 2020). Supporting the argument that more research on the recurrence of stroke events is still necessary.

Uric acid (UA), the final product of purine degradation in humans, can induce oxidative stress and promote the development of atherosclerosis and inflammation (Song and Zhao, 2018; Kimura et al., 2020). Studies have documented a close link between UA with inflammatory polyarthropathy, hypertensive disease, circulatory disease, metabolic disorders, and neurological disorders (Brody et al., 2016; Desideri et al., 2017; Li et al., 2019; Cortese et al., 2020; Ma et al., 2021). Cerebrovascular disorders are reported to be associated with UA, and the UA level has a protective effect in the prognosis of acute ischemic stroke (Wang et al., 2016; Lei et al., 2019). A tertiary analysis of the URICO-ICTUS trial showed that UA therapy could reduce the incidence of ischemic stroke exacerbations significantly (Amaro et al., 2016). while other studies have found that an elevated level of UA may contribute to lacunar infarction and carotid atherosclerosis (Crosta et al., 2018; Song and Zhao, 2018; Mannarino et al., 2021). Research has also suggested that only suitable concentrations of UA have a neuroprotective effect (Zhang et al., 2016, 2017). Thus, whether the UA level is a protective factor or a risk factor for cerebrovascular disease remains controversial.

An inflammatory response, the exact nature of which is suggested by blood inflammatory markers can occur in many diseases (Umemura et al., 2011; Mozos et al., 2017; Darweesh et al., 2018). Inflammation plays a major role in the stiffening of large arteries, and is linked to atherosclerosis, arteriosclerosis, endothelial dysfunction, and oxidative stress. Inflammatory markers have been reported to be associated with ischemic stroke recurrence, and the level of inflammatory markers at admission may have a clinical role in identifying those at a higher risk of death or recurrence (Whiteley et al., 2011; Mobarra et al., 2019). The blood neutrophil-to-lymphocyte ratio (NLR) has become a relatively popular indicator of inflammation recently (Arbel et al., 2014; Galizia et al., 2015). And it is a simple, inexpensive, and useful tool to indicate subclinical low-grade inflammation in many systemic circumstances (Taşoğlu et al., 2016). The UA level is closely related to inflammation indicators (Ruggiero et al., 2006; Kocaman et al., 2009; Liu et al., 2019; Bolayir et al., 2021); for example, a community-based study showed that inflammation indicators are positively correlated with UA, and this association is independent of the usual risk factors for chronic kidney disease (CKD) (Liu et al., 2019). However, the relationship among ischemic stroke recurrence, UA level and NLR is not clear.

This study therefore examine whether ischemic stroke recurrence is associated with the UA level and considered whether NLR plays a role in the relationship.

## Materials and Methods

### Demographic Data and Clinical Information

We collected baseline data of patients who were hospitalized in the neurology department of the First Affiliated Hospital of Wenzhou Medical University from 2015 to 2017. Patients with acute cerebral infarction confirmed by magnetic resonance imaging during hospitalization were included. In contrast, those who met any the following criteria were excluded: (1) A history of malignant tumors, autoimmune diseases, gouty arthritis, kidney disease, or systemic infection; (2) recent use of drugs related to UA metabolism; and (3) missing data. Each patient was followed up with for 3 years after the baseline period, and the recurrence of cerebral infarction was recorded. Demographic data, including age, gender, hypertension, diabetes, atrial fibrillation, coronary heart disease, valvular disease, homocysteine (HCY) concentration, carotid atherosclerosis, intracranial arteriosclerosis, peripheral arteriosclerosis, and TOAST were collected. Indicators of inflammation, such as white blood cell count (WBC), absolute neutrophil count (ANC), absolute lymphocyte count (ALC), NLR and UA level were collected. Finally, magnetic resonance imaging data were collected. Patients were stratified into three groups according to UA level, as follows: ≤ 266, 267–339, and ≥ 340 μmol/L (Hu et al., 2020). All data came from the electronic medical records system of the participating hospital. All participants signed informed consent forms before enrollment.

The serum levels of UA were measured using an automatic biochemical analyzer (AU5800; Beckman Coulter, Brea, CA, United States) in the laboratory of our hospital. Serum ALC and ANC values were also measured using an automatic blood cell analyzer (Sysmex XE-2100 from Biostad Analytical, Saint-Julie, Québec, Canada or SP-1000i from Sysmex, Kobe, Japan) in the laboratory of our hospital. And the NLR value is the ratio of ANC to ALC.

### Statistical Analysis

Data were described using mean and standard deviation values for normally distributed continuous variables and median and interquartile range values for non-normally distributed continuous variables. Frequencies with percentages were used to describe categorical variables. Baseline characteristics were compared using the chi-squared test, analysis of variance, or Mann-Whitney *U*-test, as appropriate.

To examine the association between UA level and the incidence of ischemic stroke recurrence, Cox proportional hazards models were used to calculate hazard ratios with 95% confidence intervals (CI). Then, 3 models were estimated, as follows: in model 1, no potential confounders were adjusted; in model 2, age, gender, hypertension, diabetes, atrial fibrillation, coronary heart disease, HCY concentration, carotid atherosclerosis, and TOAST were adjusted; and in model 3, the variables in model 2 and NLR were adjusted. In addition, we explored the potential non-linear associations using 3-knotted (fifth, 50th, and 95th) restricted cubic spline regression.

To further explore the associations of UA and NLR with the recurrence of ischemic stroke, we calculated the mediation proportion by the mediator (NLR) for the association of UA and the recurrence of ischemic stroke. We also performed an interaction and joint analysis of UA level and NLR with ischemic stroke recurrence.

To test the robustness and potential variations in different subgroups, we repeated the analyses stratified by age, gender, hypertension, diabetes, atrial fibrillation, coronary heart disease, and intracranial atherosclerosis.

We conducted several sensitivity analyses. First, we chose ANC and ALC to replace NLR for repeated analysis. Second, WBC was chosen to replace NLR to analyze the association between inflammation indicators and UA level in ischemic stroke recurrence. Third, we excluded participants with cardiac disease or arteriosclerosis because both UA level and NLR may be affected by these diseases.

## Results

### Baseline Characteristics

**FIGURE 1:**
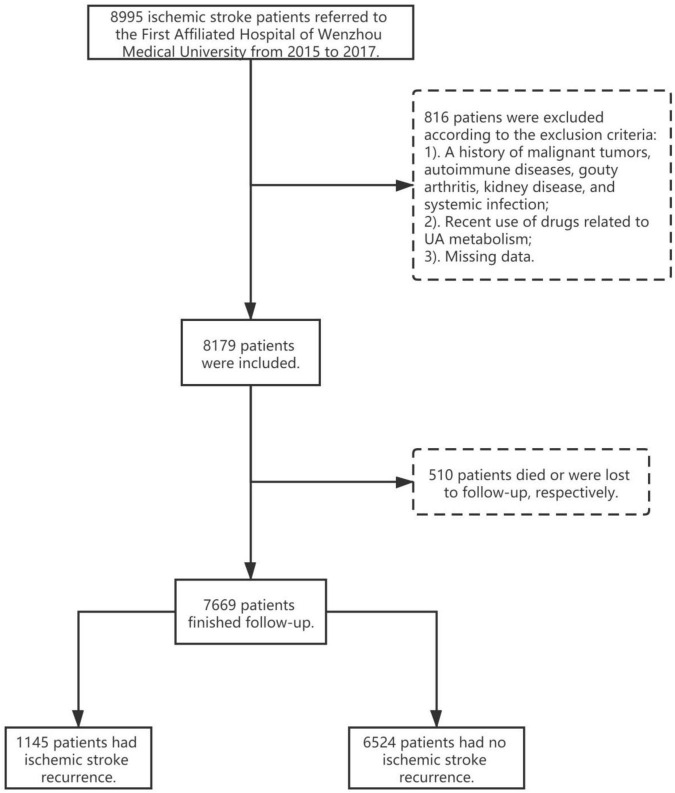
Flow chart of this research.

Supplementary Table 1 and Table 1 show the baseline characteristics of participants. Of the total 8,995 patients who had suffered an ischemic stroke, 816 patients were excluded from this study (Figure 1). Therefore, a total of 8,179 patients were enrolled in the final study, and 7,669 patients remained in the study through the follow-up period. In this study, recurring cases (*N* = 1,145; mean age, 66 years; 62.89% men) were more likely to be male and older; have a higher WBC, higher ANC, lower ALC, and higher UA level; and have hypertension, diabetes, atrial fibrillation, and atherosclerosis. Gender, hypertension, diabetes, atrial fibrillation, carotid atherosclerosis, HCY concentration, TOAST, WBC count, and NLR were significantly different (*p* ≤ 0.05 for all) among the 3 groups grouped by UA level. Patients with high UA levels tended to have high WBCs and high NLRs.

**TABLE 1:** Baseline characteristics of participants according to tertiles of UA.

Variables	Total	T1 (≤266 μmol/L)	T2 (267–339 μmol/L)	T3 (≥340 μmol/L)	*p*-value
**Baseline characteristics**					
Gender, male (%)	4,823 (62.89)	1,153 (45.43)	1,712 (66.59)	1,958 (76.48)	<0.001
Age, years	66.00 (58.00, 74.00)	66.00 (58.00, 74.00)	66.00 (58.00,74.00)	66.00 (56.00,75.00)	0.668
Hypertension, *n* (%)	5,879 (76.66)	1,906 (75.10)	1,964 (76.39)	2,009 (78.48)	0.016
Diabetes, *n* (%)	2,620 (34.16)	1,043 (41.10)	838 (32.59)	739 (28.87)	<0.001
Atrial fibrillation, *n* (%)	873 (11.38)	245 (9.65)	272 (10.58)	356 (13.91)	<0.001
Coronary heart disease, *n* (%)	229 (2.99)	58 (2.29)	84 (3.27)	87 (3.40)	0.039
Valvular disease, *n* (%)	63 (0.82)	23 (0.91)	20 (0.78)	20 (0.78)	0.846
HCY, *n* (%)	210 (2.74)	49 (1.93)	77 (2.99)	84 (3.28)	0.008
Carotid atherosclerosis, *n* (%)	2,446 (31.89)	762 (30.02)	832 (32.36)	852 (33.28)	0.037
Intracranial arteriosclerosis, *n* (%)	2,560 (33.38)	846 (33.33)	866 (33.68)	848 (33.13)	0.912
Peripheral arteriosclerosis, *n* (%)	2,881 (37.57)	914 (36.01)	988 (38.43)	979 (38.24)	0.140
**TOAST**					<0.001
1	2,965 (38.66)	982 (38.69)	1,043 (40.57)	940 (36.72)	
2	842 (10.98)	230 (9.06)	263 (10.23)	349 (13.63)	
3	1,113 (14.51)	385 (15.17)	367 (14.27)	361 (14.10)	
4	33 (0.43)	9 (0.35)	11 (0.43)	13 (0.51)	
5	2,716 (35.42)	932 (36.72)	887 (34.50)	897 (35.04)	
**Inflammation indicators**					
WBC	7.24 (6.00, 8.71)	7.02 (5.76, 8.52)	7.14 (5.98, 8.63)	7.52 (6.27, 9.03)	<0.001
ANC	0.65 (0.59, 0.72)	0.65 (0.58, 0.72)	0.65 (0.59, 0.71)	0.65 (0.59, 0.72)	0.016
ALC	0.24 (0.18, 0.30)	0.24 (0.18, 0.31)	0.24 (0.18, 0.30)	0.24 (0.18, 0.29)	<0.001
NLR	2.72 (1.97, 3.95)	2.68 (1.90, 3.94)	2.70 (1.98, 3.85)	2.78 (2.05, 4.13)	0.001
**UA**	301.00 (250.00, 360.00)	228.00 (200.00, 249.00)	301.00 (284.00, 319.00)	389.00 (360.00, 434.50)	<0.001

*Normal continuous variables were described by mean (standard deviations), skewed continuous variables were described by median (IQR), and frequencies (percentages) were used to describe categorical variables. Categorical variables were compared using the χ^2^-test. Analysis of variance was used to compare normal distribution variables. Asymmetrically distributed variables were compared with the Mann–Whitney test.*

*HCY, homocysteine; WBC, white blood cell counts; ANC, absolute neutrophil count; ALC, absolute lymphocyte count; NLR, Neutrophil-to-Lymphocyte ratio; UA, uric acid.*

### Independent Clinical Outcome Predictors: Role of Uric Acid

Table 2 showed associations of UA and NLR with the incidence of ischemic stroke recurrence. Patients with high UA levels had a higher risk of recurrence (*p* for trend < 0.0001). As shown in Table 2, after adjusting for potential confounders, compared to the baseline group (UA level ≤ 266 μmol/L), patients with a higher (≥ 340 μmol/L) or moderate (267–339 μmol/L) UA level were 1.4–2 times more likely to have ischemic stroke recurrence [odds ratio (OR), 1.432; 95% CI, 1.220–1.682; and OR, 1.965; 95% CI, 1.681–2.297, respectively]. Furthermore, after adjusting for NLR, the trend persisted, but the recurrence risk ratio changed. The above results were not materially changed in sensitivity analyses or subgroup analysis (Supplementary Table 2 and Supplementary Figure 1). From these finding, we inferred that there is an association between UA level and ischemic stroke recurrence, which may be affected by NLR.

**TABLE 2:** Associations of UA with incident of ischemic stroke recurrence.

Tertiles	Model 1		Model 2		Model 3	
	HR (95% CI)	*p*-value	HR (95% CI)	*p*-value	HR (95% CI)	*p*-value
T1	1 (reference)		1 (reference)	–	1 (reference)	–
T2	1.406 (1.200, 1.647)	<0.0001	1.432 (1.220, 1.682)	<0.0001	1.470 (1.252, 1.727)	<0.0001
T3	1.972 (1.697, 2.291)	<0.0001	1.965 (1.681, 2.297)	<0.0001	1.926 (1.648, 2.250)	<0.0001
P for trend		<0.0001		<0.0001		<0.0001

*Model 1, no potential confounders were adjusted; Model 2, age, gender, hypertension, diabetes, atrial fibrillation, coronary heart disease, HCY, carotid atherosclerosis, TOAST were adjusted; Model 3, adjusted for covariates from Model 2 and further adjusted for NLR.*

*CI, confidence interval; HR, hazard ratio.*

**FIGURE 2:**
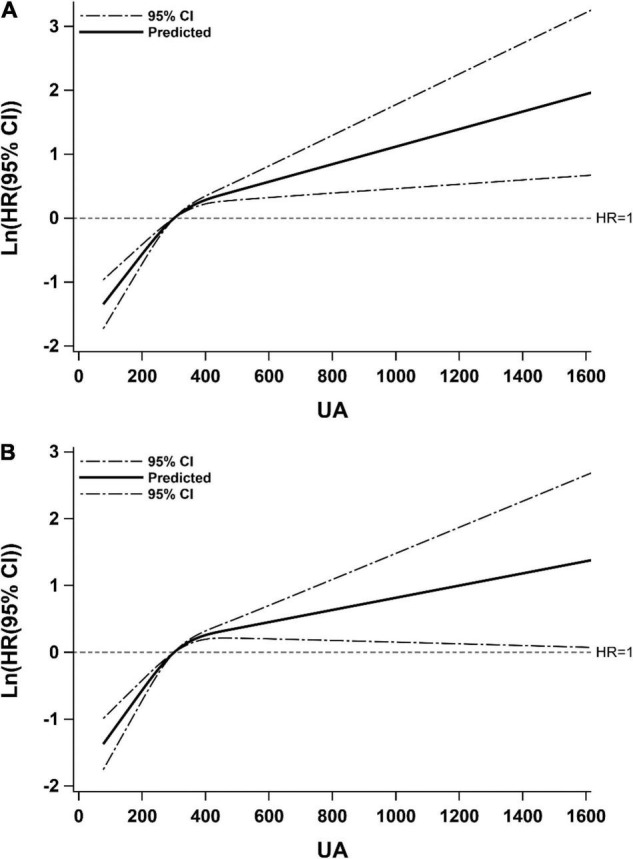
Restricted cubic spline for associations of UA with ischemic stroke recurrence. Model **(A)** adjusted for age, gender, hypertension, diabetes, atrial fibrillation, coronary heart disease, HCY, carotid atherosclerosis, TOAST; Model **(B)** adjusted for covariates from Model A and further adjusted for NLR. Hazard ratios were indicated by solid lines and 95% CIs by dotted lines. CI, confidence interval; HR, hazard ratio; UA, uric acid.

In Figure 2, we used restricted cubic splines to flexibly model and visualize the relationship of predicted UA level and the recurrence of ischemic stroke. It was found that there was a non-linear relationship between UA level and recurrence: specifically, the risk of recurrence increased rapidly with a rise in UA level to 300 μmol/L, then slowed down. What’s more, the relationship may be influenced by adjusting for NLR.

### Analysis of the Mediating Effect of Neutrophil-to-Lymphocyte Ratio on the Association of Uric Acid and Ischemic Stroke Recurrence

To explore whether NLR is a mediating factor in the relationship between the increase in UA level and ischemic stroke recurrence, a mediating effect analysis was performed. As seen in Table 3, we found that NLR may be a partial mediating factor, but its mediating effect is not strong. Meanwhile, when comparing models 1 and 2, we found that there may be an interaction between UA level and NLR level.

**TABLE 3:** Mediation analysis of NLR on associations of UA with ischemic stroke recurrence.

	NDE	NIE	TE	Estimated percent mediated (%)
Model 1	1.0033 (1.0025, 1.0040)	1.0002 (1.0001, 1.0003)	1.0035 (1.0027, 1.0042)	5.0906 (2.4953, 7.6860)
Model 2	1.0033 (1.0026, 1.0040)	1.0002 (1.0001, 1.0003)	1.0035 (1.0027, 1.0042)	5.2109 (2.5575, 7.8642)

*Model 1, no interaction terms; Model 2, include interaction terms with UA and NLR. Adjusted for age, gender, hypertension, diabetes, atrial fibrillation, coronary heart disease, HCY, carotid atherosclerosis, TOAST.*

*NDE, natural direct effect; NIE, natural indirect effect; TE, total effect.*

### Interaction and Joint Analysis of Neutrophil-to-Lymphocyte Ratio and Uric Acid Level in Ischemic Stroke Recurrence

**FIGURE 3:**
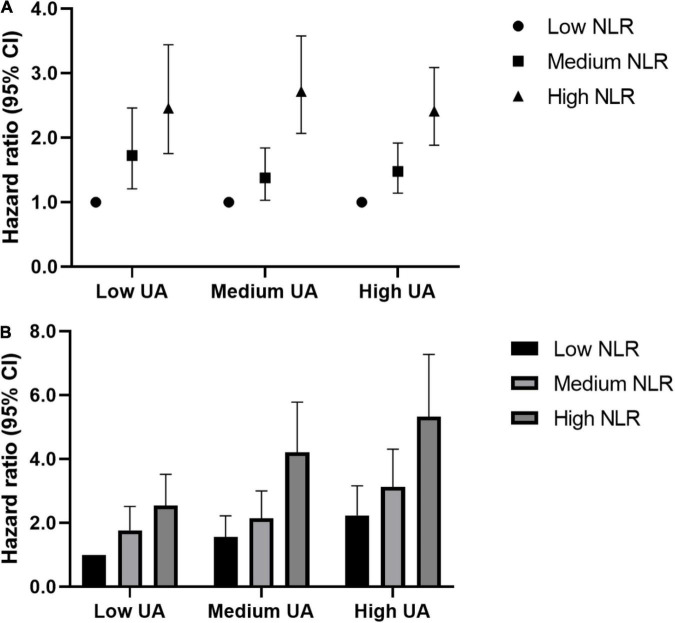
Interaction and joint associations of UA and NLR with ischemic stroke recurrence. Hazard ratios were adjusted for age, gender, hypertension, diabetes, atrial fibrillation, coronary heart disease, HCY, carotid atherosclerosis, TOAST. **(A)** Multiplicative interaction was evaluated using hazard ratios for the product term between the NLR (low v high) and UA (low v high), and the multiplicative interaction was statistically significant when its confidence interval did not include 1. Additive interaction was evaluated using relative excess risk due to interaction (RERI) between the NLR (low v high) and UA (low v high), and the additive interaction was statistically significant when its confidence interval did not include 0. **(B)** The highest risks of ischemic stroke recurrence were seen in adults of high SUA and high NLR, which had a 433% increased risk of ischemic stroke recurrence [HR (95%CI): 5.33 (3.91,7.27)], compared to the same reference group (low UA and low NLR). NLR, Neutrophil-to-Lymphocyte ratio; UA, uric acid.

To further explore the relationship between NLR and UA in the recurrence of ischemic stroke, an interaction and joint analysis was performed. We also divided the NLR level into 3 categories. As seen in Figure 3A, we found that there was an additive interaction (relative excess risk due to interaction, 1.05; 95% CI, 0.48–1.62) and no multiplicative interaction (hazard ratio for the product term, 1.04; 95% CI, 0.73–1.48) between the UA and NLR level in ischemic stroke recurrence. And as seen in Figure 3B, the greatest risk of ischemic stroke recurrence was present in patients with high UA levels and high NLRs, who exhibited a 433% increased risk of ischemic stroke recurrence (hazard ratio, 5.33; 95% CI, 3.91–7.27) compared to the reference group (with a low UA level and low NLR).

## Discussion

### Summary of the Main Findings

First, our research showed that UA is a risk factor for the recurrence of ischemic stroke in older adults. Second, UA is non-linearly associated with ischemic stroke recurrence. Third, there is an impact of NLR on the association between UA and ischemic stroke recurrence. In other words, there is a correlation between UA level and stroke recurrence, and NLR has an additive role in this relationship. Thus, we speculate that a high UA level may induce ischemic stroke recurrence through inflammatory mechanisms.

### Prior Research and How Our Study Differs

Numerous studies have shown that UA level is associated with cerebrovascular disease, but whether it is a protective factor or worsens the risk of developing cerebrovascular disease remains controversial. This study found that there is an association between UA and the recurrence of ischemic stroke, and the possible mechanisms are: (1) Oxidation and anti-oxidation and (2) Inflammation.

Many studies have shown that UA can promote oxidative stress (Packer, 2020). UA may contribute to increased oxidative stress independent of xanthine oxidoreductase activity by increasing reactive oxygen species (ROS) production, without affecting ROS scavenging (Kurajoh et al., 2021). Research has confirmed that UA can promote oxidation through chronic inflammation (Inaba et al., 2013). In addition, studies have shown that UA can exacerbate atherosclerosis (Song and Zhao, 2018; Kimura et al., 2020). What’s more, a meta-analysis found that a high concentration of UA is related to the thickness of the carotid artery intima-media (Ma et al., 2021). And there is no disputing of the deleterious effects and detrimental contribution of oxidative stress to lesion progression following ischemic stroke (Shirley et al., 2011; Zhang et al., 2011).

However, studies have also shown that UA has anti-oxidative stress and neuroprotective effects. UA is a powerful antioxidant and a scavenger of singlet oxygen and radicals. It has antioxidant activity, can scavenge ROS and protects cells from oxidative stress (Ames et al., 1981; Sun et al., 2017). In addition, the appropriate concentration of UA has a neuroprotective effect, so the degree of brain damage and the generation of ROS are reduced after adding an appropriate amount of UA (Zhang et al., 2017). And UA was demonstrated to protect dopaminergic neurons in Parkinson’s mice through the modulation of neuro-inflammation and oxidative stress (Huang et al., 2017).

From the above studies, we surmise that the role of UA in cerebrovascular disease remains controversial. We speculate that the reasons may be as follows: (1) the research population, research objectives, and research methods are inconsistent; (2) UA stratification methods are inconsistent; and (3) the sample sizes of previous studies were not enough. Therefore, our research sought to further explore the role of UA in the recurrence of ischemic stroke. We found that UA is a risk factor for the recurrence of ischemic stroke, and patients with high levels of UA may have a 92.6% increased risk of recurrence.

UA also can induce inflammation. A large population-based sample of elderly and normal UA participants showed that UA has a significant positive correlation with inflammatory markers, such as C-reactive protein and interleukin-6 (Ruggiero et al., 2006). Studies have shown that UA stimulates the expression of C-reactive protein, fibrinogen, ferritin, and complement C3, and induces inflammation by activating the nuclear factor kappa-light-chain-enhancer of activated B-cells (NF-κB) signaling pathway in HepG2 cells (Spiga et al., 2017). One study found that a high concentration of UA (up to 50 mg/dL) can strengthen the enhanced state of chronic inflammation by altering the balance of interleukin-1β/interleukin-1Ra (Crişan et al., 2016). Another study found that UA increases the release of ROS, depolarizes the mitochondrial membrane potential, increases the expression levels of TLR4 and NLRP3, and activates the NLRP3 inflammasome and NF-κB signaling to induce inflammation (Ma et al., 2020). What’s more, UA also can induce inflammation through the AMP-activated protein kinase–mTOR (mammalian target of rapamycin) mitochondrial ROS and hypoxia-inducible factor-1α pathway (Kimura et al., 2020). Inflammatory markers, such as C-reactive protein, interleukin-6, tumor necrosis factor, and fibrinogen, are up-regulated following acute stroke. The associations of these biomarkers with increased mortality, recurrent vascular risk, and poor functional outcome of stroke have been reported (Whiteley et al., 2011; Mobarra et al., 2019; Kirzinger et al., 2021). Therefore, we speculate that UA may cause recurrence of ischemic stroke through inflammation.

The NLR is a novel biomarker that can single out individuals at risk for vascular events (Arbel et al., 2014). NLR has been found to be associated with many diseases, such as cancer, cardiovascular diseases, and CKD (Im et al., 2013; Kocyigit et al., 2013; Ebata et al., 2021). Studies have demonstrated that NLR is significantly associated with clinical outcomes in acute ischemic stroke (AIS). Meta-analyses have shown that an elevated NLR is associated with ischemic stroke severity, hemorrhagic transformation, and poor clinical outcomes, suggesting adverse effects of stroke-related inflammation (Song et al., 2019; Zhang et al., 2019). A prospective study found that a low NLR level was strongly associated with the degree of reperfusion following mechanical stroke thrombectomy in acute ischemic stroke patients, suggesting that a low NLR level could reduce the risks of symptomatic intracranial hemorrhage and mortality (Aly et al., 2021). Through these studies, we can draw the conclusion that NLR has an important impact on ischemic stroke, reminding us to focus on NLR in stroke patients.

In recent years, the relationship between UA and NLR has become a research hot-spot. Use of NLR as an indicator of inflammation is very valuable, and it can be evaluated by a simple blood count analysis. One study showed that UA is positively correlated with NLR, and NLR may be a determinant of inflammation and atherosclerosis in CKD patients (Yilmaz et al., 2017). A Chinese cohort study of CKD also confirmed that NLR is associated with the risk of end-stage renal disease, suggesting that NLR can be used for end-stage renal disease risk assessment in advanced CKD patients (Yuan et al., 2019). However, the relationship between UA and NLR remains controversial. For example, in a study of multiple sclerosis, NLR was found to be negatively correlated with UA (Bolayir et al., 2021). The same study also found that co-assessment of NLR and UA may be more effective than assessing these parameters individually in demonstrating disability in multiple sclerosis patients. From these studies, it can be seen that there is a relationship between UA level and HLR, but it may be inconsistent in different diseases. This also attracted us to explore the relationship further.

From the above discussion and analysis, we drew a conclusion that both UA and NLR are closely related to ischemic stroke, with a circular relationship existing among all 3 items. However, few studies to date have looked at UA, NLR, and stroke recurrence together.

Therefore, this study explored the correlation between NLR and UA in the recurrence of ischemic stroke, and we found that NLR has a mediating effect in the association between UA and ischemic stroke recurrence. Although the effect is not strong, there was an additive interaction between NLR and UA.

### Strengths and Limitations

Many previous studies have revealed the relationship between UA and the outcomes of ischemic stroke (Wang et al., 2016; Lei et al., 2019), but there are few investigations of the correlation between UA and ischemic stroke recurrence, and no studies have assessed the relationship among UA, NLR, and ischemic stroke recurrence simultaneously. This is the innovative point and a major strength of this research. What’s more, the advantages of this study include that the sample size is large and sensitivity and subgroup analyses were performed. In contrast, the limitations are as follows: (1) this study was a single-center study, and more cohorts are required for verification; (2) some participants were confirmed to have ischemic stroke recurrence by telephone, so there may be missed diagnoses; (3) although blood samples were all collected during the first episode of hospitalization, but there were also inconsistencies in the timing of blood collection. Future research needs to pay attention to these limitations.

## Conclusion

Thus, our research provides evidence that high concentrations of UA promote ischemic stroke recurrence in older adults. This study confirms the non-linear relationship between UA and the recurrence of ischemic stroke and suggests that this relationship may be affected by NLR. This study reminds clinicians to pay attention to UA level and inflammation in ischemic stroke patients, which may help to prevent the recurrence of ischemic stroke.

## Data Availability Statement

The raw data supporting the conclusions of this article will be made available by the authors, without undue reservation.

## Ethics Statement

The studies involving human participants were reviewed and approved by the Institutional Review Board and Research Ethics Committee of The First Affiliated Hospital of Wenzhou Medical University. The patients/participants provided their written informed consent to participate in this study.

## Author Contributions

H-YZ collected, analyzed the data, interpreted the data, and drafted the manuscript. S-ZZ prepared the figures and analyzed the data. M-LZ collected the data and prepared the tables. YW prepared the figures and collected the data. Z-MP collected the data. H-RC helped to draft the manuscript. KZ obtained funding and helped to revise the manuscript. ZW designed the study, obtained funding, and helped to draft the manuscript. All authors saw and approved the final version of the manuscript.

## Conflict of Interest

The authors declare that the research was conducted in the absence of any commercial or financial relationships that could be construed as a potential conflict of interest.

## Publisher’s Note

All claims expressed in this article are solely those of the authors and do not necessarily represent those of their affiliated organizations, or those of the publisher, the editors and the reviewers. Any product that may be evaluated in this article, or claim that may be made by its manufacturer, is not guaranteed or endorsed by the publisher.
