# Supplementary material for: Elevated Serum Uric Acid Increases the Risk of Ischemic Stroke Recurrence and Its Inflammatory Mechanism in Older Adults
Source: Front Aging Neurosci. 2022 Mar 8;14:822350. doi: 10.3389/fnagi.2022.822350 (PMC8957925; doi:10.3389/fnagi.2022.822350)
Supplement: Supplementary file 1 [file Data_Sheet_1.docx]

**Supplemental Material**

Table S1 Baseline characteristics of participants according to recurrence.

| **Variables** | **Total** | **No recurrence** | **Recurrence** | **P value** |
| --- | --- | --- | --- | --- |
| **Baseline characteristics** |  |  |  |  |
| Gender, male (%) | 4823(62.89) | 4047(62.03) | 776(67.77) | <0.001 |
| Age, years | 66.00(58.00,74.00) | 66.00(57.00,74.00) | 69.00(61.00,76.00) | <0.001 |
| Hypertension, n (%) | 5879(76.66) | 4937(75.67) | 942(82.27) | <0.001 |
| Diabetes, n (%) | 2620(34.16) | 2098(32.16) | 522(45.59) | <0.001 |
| Atrial fibrillation, n (%) | 873(11.38) | 723(11.08) | 150(13.10) | 0.047 |
| Coronary heart disease, n (%) | 229(2.99) | 186(2.85) | 43(3.76) | 0.097 |
| Valvular disease, n (%) | 63(0.82) | 53(0.81) | 10(0.87) | 0.833 |
| HCY, n (%) | 210(2.74) | 144(2.21) | 66(5.76) | <0.001 |
| Carotid atherosclerosis, n (%) | 2446(31.89) | 1982(30.38) | 464(40.52) | <0.001 |
| Intracranial arteriosclerosis, n (%) | 2560(33.38) | 2081(31.90) | 479(41.83) | <0.001 |
| Peripheral arteriosclerosis, n (%) | 2881(37.57) | 2321(35.58) | 560(48.91) | <0.001 |
| **TOAST** |  |  |  | <0.001 |
| 1 | 2965(38.66) | 2498(38.29) | 467(40.79) |  |
| 2 | 842(10.98) | 695(10.65) | 147(12.84) |  |
| 3 | 1113(14.51) | 899(13.78) | 214(18.69) |  |
| 4 | 33(0.43) | 27(0.41) | 6(0.52) |  |
| 5 | 2716(35.42) | 2405(36.86) | 311(27.16) |  |
| **Inflammation indicators** |  |  |  |  |
| WBC | 7.24(6.00,8.71) | 7.14(5.92,8.59) | 7.73(6.44,9.54) | <0.001 |
| ANC | 0.65(0.59,0.72) | 0.65(0.58,0.71) | 0.69(0.63,0.76) | <0.001 |
| ALC | 0.24(0.18,0.30) | 0.25(0.19,0.31) | 0.20(0.15,0.26) | <0.001 |
| NLR | 2.72(1.97,3.95) | 2.63(1.91,3.76) | 3.35(2.41,5.29) | <0.001 |
| **UA** | 301.00(250.00,360.00) | 297.00(246.00,355.00) | 328.00(272.00,384.00) | <0.001 |

Notes: Normal continuous variables were described by mean (standard deviations), skewed continuous variables were described by median (IQR), and frequencies (percentages) were used to describe categorical variables. Categorical variables were compared using the χ2 test. Analysis of variance was used to compare normal distribution variables. Asymmetrically distributed variables were compared with the Mann–Whitney test.

Abbreviations: HCY, homocysteine; WBC, white blood cell counts; ANC, absolute neutrophil count; ALC, absolute lymphocyte count; NLR, Neutrophil-to-Lymphocyte ratio; UA, uric acid.

**Subgroup analyses**

Stratified by age, gender, hypertension, diabetes，atrial fibrillation, coronary heart disease, Intracranial arteriosclerosis.

| 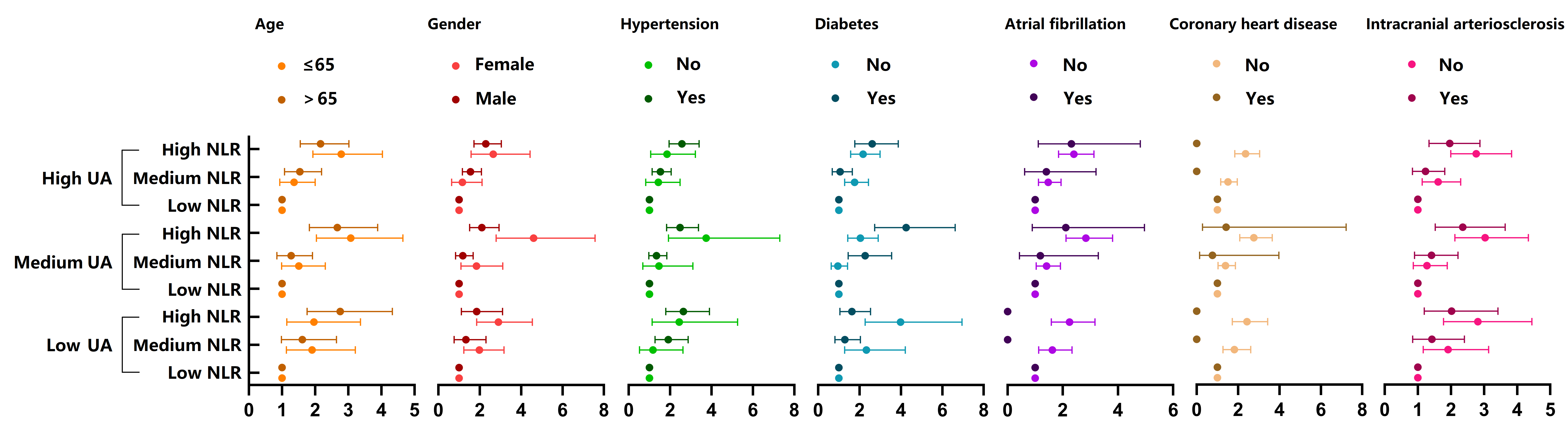 |
| --- |
| Figure S1. Association of the NLR with ischemic stroke of recurrence by UA: subgroup analyses.  Notes: Hazard ratios were adjusted for age, gender, hypertension, diabetes, atrial fibrillation, coronary heart disease, Intracranial atherosclerosis. The results of the main analysis did not change substantially in the subgroup analysis.  Abbreviations: NLR, Neutrophil-to-Lymphocyte ratio; UA, uric acid. |

**Sensitivity analyses**

Table S2. Associations of UA with incident of ischemic stroke recurrence: sensitivity analyses.

| **Analyses** | **Unadjusted** | |  | **Adjusted** | |
| --- | --- | --- | --- | --- | --- |
|  | **Hazard ratio (95% CI)** | **p value** |  | **Hazard ratio (95% CI)** | **p value** |
| Main analyses ^a^ |  |  |  |  |  |
| T1 | 1 (reference) | - |  | 1 (reference) | - |
| T2 | 1.432(1.220,1.682) | <0.0001 |  | 1.470(1.252,1.727) | <0.0001 |
| T3 | 1.965(1.681,2.297) | <0.0001 |  | 1.926(1.648,2.250) | <0.0001 |
| p for trend |  | <0.0001 |  |  | <0.0001 |
| Adjusted for ANC ^b^ | |  |  |  |  |
| T1 | 1 (reference) | - |  | 1 (reference) | - |
| T2 | 1.432(1.220,1.682) | <0.0001 |  | 1.448(1.233,1.700) | <0.0001 |
| T3 | 1.965(1.681,2.297) | <0.0001 |  | 1.934(1.656,2.258) | <0.0001 |
| p for trend |  | <0.0001 |  |  | <0.0001 |
| Adjusted for ALC ^c^ | |  |  |  |  |
| T1 | 1 (reference) | - |  | 1 (reference) | - |
| T2 | 1.432(1.220,1.682) | <0.0001 |  | 1.465(1.248,1.720) | <0.0001 |
| T3 | 1.965(1.681,2.297) | <0.0001 |  | 1.946(1.666,2.273) | <0.0001 |
| p for trend |  | <0.0001 |  |  | <0.0001 |
| Adjusted for ANC and ALC ^d^ | |  |  |  |  |
| T1 | 1 (reference) | - |  | 1 (reference) | - |
| T2 | 1.432(1.220,1.682) | <0.0001 |  | 1.456(1.240,1.709) | <0.0001 |
| T3 | 1.965(1.681,2.297) | <0.0001 |  | 1.938(1.660,2.264) | <0.0001 |
| p for trend |  | <0.0001 |  |  | <0.0001 |
| Adjusted for WBC ^e^ | |  |  |  |  |
| T1 | 1 (reference) | - |  | 1 (reference) | - |
| T2 | 1.432(1.220,1.682) | <0.0001 |  | 1.415(1.206,1.662) | <0.0001 |
| T3 | 1.965(1.681,2.297) | <0.0001 |  | 1.863(1.594,2.178) | <0.0001 |
| p for trend |  | <0.0001 |  |  | <0.0001 |
| Excluding individuals with cardiac disease ^f^ | |  |  |  |  |
| T1 | 1 (reference) | - |  | 1 (reference) | - |
| T2 | 1.404(1.181,1.670) | <0.0001 |  | 1.450(1.219,1.725) | <0.0001 |
| T3 | 1.948(1.646,2.306) | <0.0001 |  | 1.939(1.639,2.294) | <0.0001 |
| p for trend |  | <0.0001 |  |  | <0.0001 |
| Excluding individuals with atherosclerosis ^g^ | |  |  |  |  |
| T1 | 1 (reference) | - |  | 1 (reference) | - |
| T2 | 1.606(1.196,2.158) | 0.002 |  | 1.723(1.280,2.319) | <0.0001 |
| T3 | 2.639(1.991,3.498) | <0.0001 |  | 2.651(2.002,3.510) | <0.0001 |
| p for trend |  | <0.0001 |  |  | <0.0001 |

Notes: The results of the main analysis did not change substantially in the sensitivity analysis. ^a^: adjusted for age, gender, hypertension, diabetes, atrial fibrillation, coronary heart disease, HCY, carotid atherosclerosis, TOAST, NLR; ^b^: adjusted for age, gender, hypertension, diabetes, atrial fibrillation, coronary heart disease, HCY, carotid atherosclerosis, TOAST, ANC; ^c^: adjusted for age, gender, hypertension, diabetes, atrial fibrillation, coronary heart disease, HCY, carotid atherosclerosis, TOAST, ALC; ^d^: adjusted for age, gender, hypertension, diabetes, atrial fibrillation, coronary heart disease, HCY, carotid atherosclerosis, TOAST, ANC, ALC; ^e^: adjusted for age, gender, hypertension, diabetes, atrial fibrillation, coronary heart disease, HCY, carotid atherosclerosis, TOAST, WBC; ^f^: adjusted for age, gender, hypertension, diabetes, HCY, carotid atherosclerosis, TOAST, NLR; ^g^: adjusted for age, gender, hypertension, diabetes, atrial fibrillation, coronary heart disease, HCY, TOAST, NLR.

Abbreviations: ANC, absolute neutrophil count; ALC, absolute lymphocyte count; HCY, homocysteine; WBC, white blood cell counts.
